# Predictors of aortic dilation in patients with coarctation of the aorta: evaluation with dual-source computed tomography

**DOI:** 10.1186/s12872-018-0863-8

**Published:** 2018-06-22

**Authors:** Qin Zhao, Ke Shi, Zhi-gang Yang, Kai-yue Diao, Hua-yan Xu, Xi Liu, Ying-kun Guo

**Affiliations:** 1 Department of Radiology, West China Hospital, Sichuan University, 37# Guo Xue Xiang, Chengdu, 610041 Sichuan China; 2 Department of Radiology, West China Second University Hospital, Sichuan University, 20# Section 3 South Renmin Road, Chengdu, 610041 Sichuan China

**Keywords:** Coarctation of the aorta, Aortic dilation, Aorta, Dual-source computed tomography, Degree of coarctation

## Abstract

**Background:**

Coarctation of aorta (CoA) may progressively develop aortic dilation at other site of the aorta and can lead to fatal aortic diseases. We aimed to evaluate the occurrence of aortic dilation and related predictors in patients with CoA using dual-source computed tomography (DSCT).

**Methods:**

Fifty-three patients with CoA identified by DSCT were retrospectively reviewed. Aortic diameters were measured at six different levels and standardized as z-scores based on the square root of body surface area. Coarctation site–diaphragm ratio (CDR) was used to describe the degree of narrowing. A total of 26 patients were included in mild group (CDR > 50%) and 27 in severe group (CDR < 50%) according to the severity of coarctation. Student’s t-test and Spearman correlation coefficients, univariate and multivariable logistic regression analyses were used to assess the risk factors including age, degree of narrowing and other malformations for aortic dilation.

**Results:**

Severe group had significantly larger z-scores of ascending aorta (2.41 ± 0.39 vs. 2.10 ± 0.57, *p* < 0.05) and post-coarctation aorta (2.17 ± 0.48 vs. 1.68 ± 0.43, *p* < 0.001) compared with mild group. Degree of coarctation was associated with the z-scores of the ascending aorta (*r* = − 0.356, *p* < 0.05) and post-coarctation aorta (*r* = − 0.414, *p* < 0.05). Collateral circulation was related to the z-scores of ascending aorta (*r* = 0.375, *p* < 0.05). Increased severity of coarctation was independent predictor of ascending (odds ratio 7.46; 95% CI 1.19–46.76; *p* < 0.05) and post-coarctation aortic dilation(odds ratio 8.42; 95% CI 1.84–38.56; *p* < 0.05).

**Conclusions:**

Ascending and post-coarctation aortic diameters or dilations were both associated with the degree of coarctation. By comprehensively evaluating the aortic diameters and associated malformations including collateral circulation, DSCT can aid in stratification of risk for aortic dilation in patients with CoA.

**Electronic supplementary material:**

The online version of this article (10.1186/s12872-018-0863-8) contains supplementary material, which is available to authorized users.

## Background

Coarctation of aorta (CoA) is one of the most common congenital heart diseases and covers about 6–8% in live births with congenital heart diseases [1]. Patients with progressive course may develop aortic dilation and are at risk for aortic diseases, including aortic aneurysm, aortic dissection and aortic rupture, which may induce sudden death [2, 3]. In recent years, postoperative progressive aortic dilation among patients with CoA has been frequently reported, but the mechanism remains unclear. Thus, evaluating the preoperative aortic diameters and exploring related factors for aortic dilation in patients with CoA are necessary.

Numerous methods have been used to evaluate the aorta in patients with CoA. Transthoracic echocardiography (TTE) is the first-line imaging modality. However, it is limited in evaluating the aorta due to its poor imaging window and difficulty in assessing collateral circulation. Conventional cardiac catheterization is the gold standard for assessing the aorta, but the invasive nature restricts its preoperative use. Magnetic resonance imaging (MRI) has gained widespread acceptance in the field, especially considering its advantage of no radiation exposure, but its high cost, long examination time and the relatively lower spatial resolution compared with CT (computed tomography) restrain the application. As a noninvasive imaging technology, CT has high spatial resolution and a wide field of view, which can help describe the aorta comprehensively. With decreased radiation exposure, dual-source CT (DSCT) has been extensively used in evaluating congenital heart diseases [4–8]. To the best of our knowledge, studies on predictors of aortic dilation in patients with CoA assessed by DSCT are rare. Therefore, this study aimed to evaluate the occurrence of aortic dilation and related predictors by DSCT in unrepaired patients with CoA.

## Methods

### Study population

Between August 2010 and September 2017, 82 patients with CoA from our hospital were retrospectively reviewed. The inclusion criteria were patients with CoA who were identified by DSCT and underwent TTE before surgery or percutaneous intervention. The exclusion criteria were patients with insufficient clinical information, other aortic diseases such as interrupted aortic arch, aortic dissection, atherosclerosis, arteritis and supravalvular aortic stenosis, malformations like Turner syndrome and Marfan syndrome, or history of cardiovascular catheter or surgical interventions. Finally, 53 patients remained (34 men and 19 women; mean age: 18.15 ± 18.48 years; range: 0.07–84 years). The institutional review board of our hospital approved our current study (No. 14–163) and granted to waive the patient consent based on the retrospective nature of the current study.

### DSCT

DSCT was performed using a related scanner (Somatom Definition; Siemens Medical Solutions, Forchheim, Germany), and a retrospectively ECG-gated protocol was used based on the following parameters: tube voltage of 80–120 kV, tube current of 100–220 mAs, gantry rotation time of 0.28–0.33 s, and pitch of 0.2–0.5. Patients younger than 6 years old were administered with a short-term sedative (chloral hydrate with 10% concentration, 0.5 mL/kg) before the cardiac DSCT examinations. Older patients were instructed to hold their breath when scanning. The scan was conducted in the craniocaudal direction, from the inlet of the thorax to 2 cm below the level of the diaphragm. Nonionic contrast agent (iopamidol, 370 mg/mL, Bracco, Italy) was injected to all patients via an antecubital vein, at a rate of 1.2–2.5 mL/s. All imaging data were analyzed on a workstation (Syngo; Siemens Medical System, Forchheim, Germany). The reconstructed images were based on a slice thickness of 0.75 mm and an increment of 0.7 mm.

### Trans-thoracic echocardiography

All patients received a standard TTE examination based on a ultrasound system (iE33; Philips Medical Systems NA, Bothell, WA, USA). Following the recommendations of the American Society of Echocardiography Committee, the examination was performed with M-mode, two-dimensional, continuous wave and Doppler color flow imaging [9].

### Image analysis

**Fig. 1:**
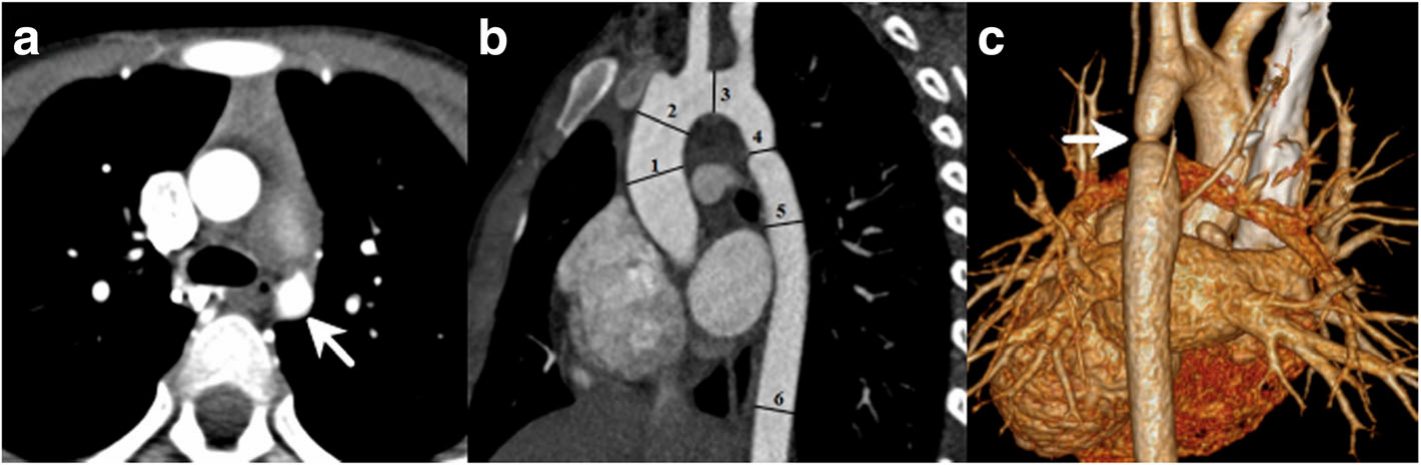
CT images of a 10-year-old man with coarctation of the aorta. **a** Axial image shows the significant stenosis of aortic isthmus (white arrow). **b** Sagittal multiplanar reformatted image shows the measurement of aortic diameters at different levels: 1, ascending aorta; 2, pre-coarctation aorta; 3, aortic arch; 4, site of coarctation; 5, descending aorta after the site of coarctation; 6, descending aorta at level of diaphragm. **c** Volume rendering image shows a short segment aortic narrowing (white arrow) below the left subclavian artery

**Fig. 2:**
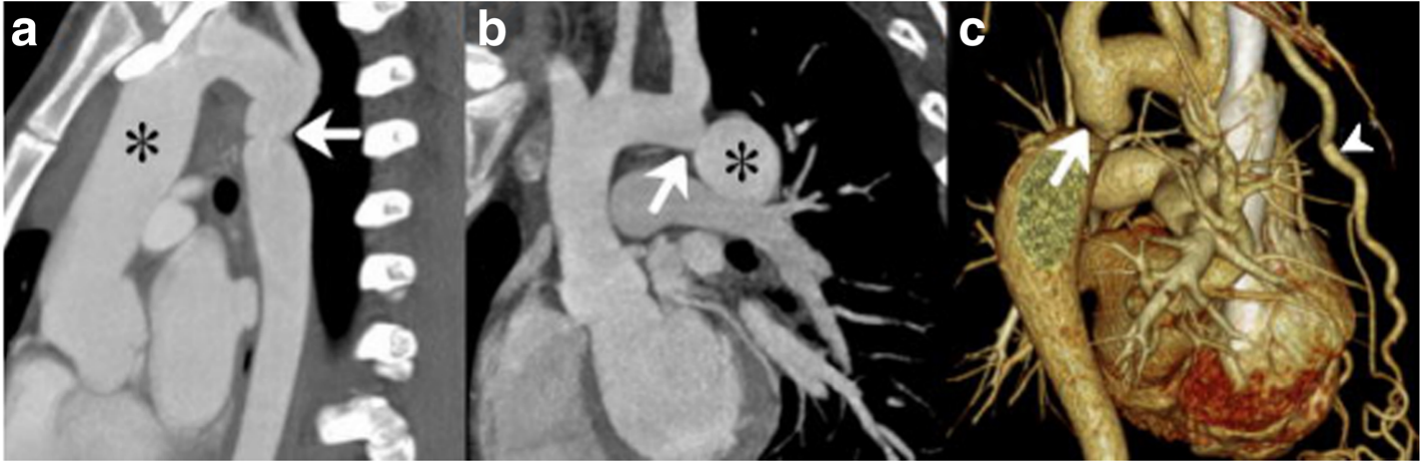
CT images of vascular abnormalities in CoA patients. Sagittal multiplanar reformatted image (**a**) shows a 16-year-old woman with CoA associated with dilated ascending aorta (asterisk) before the narrowing (white arrow). Sagittal multiplanar reformatted image (**b**) shows a 19-year-old man with CoA associated with dilated descending aorta (asterisk) after the narrowing (white arrow). Volume rendering image (**c**) shows a 53-year-old CoA woman with the narrowing (white arrow) and collateral circulation including enlarged internal mammary artery (arrowhead). Aortic dilation was considered if the z-score > 2

The aortic diameters and concomitant cardiac malformations including collateral circulation, bicuspid aortic valve (BAV), patent ductus arteriosus (PDA), ventricular septal defect (VSD), atrial septal defect (ASD), aortic regurgitation (AR) and aortic valve stenosis (AS) were recorded. The axial images, maximum intensity projection and volume rendering were used to analyze images by two experienced radiologists. Aortic diameters were measured at six levels: ascending aorta at its maximum diameter (ascending aorta), aorta just proximal to the origin of the brachiocephalic trunk (pre-coarctation aorta), aortic arch at the largest size (aortic arch), coarctation site at the narrowest size (coarctation site), the widest region of the descending aorta (post-coarctation aorta), descending aorta at the level of the diaphragm (Fig. 1) [10, 11]. The ratio of the aortic diameter at the coarctation site to that at the diaphragm (coarctation site–diaphragm ratio, CDR) was calculated to describe the degree of coarctation. The CoA was identified with a CDR less than 75% [2]. Patients with CoA were classified into two groups based on the severity of coarctation: mild group (CDR > 50%) and severe group (CDR < 50%) [2]. Given the growth-related changes, aortic diameter was standardized as z-score, the ratio of the aortic diameters over the square root of body surface area (BSA) [12]. Aortic dilation was considered if the z-score > 2 (Fig. 2) [12]. Age, BSA, gender, complexity of CoA, presence of BAV, PDA, VSD, AR, AS, history of hypertension (blood pressure > 140/90 mmHg) were assessed as aortic diameter associated factors. Complex CoA was defined as CoA with other cardiovascular abnormalities.

### Radiation dose estimation

After DSCT examination, volume CT dose index and dose-length product were automatically shown on the CT console. The effective dose was calculated using conversion coefficients referring to the 2007 recommendations of the International Commission on Radiological Protection [13, 14].

### Statistical analysis

Statistical analysis was conducted with SPSS software for Windows (version 24.0, SPSS Inc., Chicago, IL, USA). All continuous variables were shown as mean ± standard deviations, and categorical variables were presented as numbers and percentages. Student’s t-test was used to compare the continuous variables, and Fischer’s exact test was performed to test the qualitative variables. The correlation between the aorta and factors was evaluated by Spearman correlation coefficients. Univariate and multivariable logistic regression analyses were performed to identify variables correlated with the occurrences of ascending and post-coarctation dilations. Candidate variables included age, gender, severity of CoA (mild/severe coarctation), history of hypertension, BAV, PDA, VSD, AR, AS, and collateral circulation. Multivariable logistic model was developed using forward stepwise method (stepping criteria: F probability < 0.05 for entry, > 0.1 for removal). Statistical significance was set at a two-tailed *p* value < 0.05.

## Results

### Patients’ characteristics

This study included 26 (49.1%) mild and 27 (50.9%) severe coarctation patients. A total of 76 cardiovascular complications were confirmed, including 21 collateral circulations (Table 1). The patients in mild group presented smaller BSA (0.85 ± 0.50 vs. 1.26 ± 0.55 m^2^, *p* < 0.05) and less collateral circulation (23.1% vs. 55.5%, *p* < 0.05) than those in the severe group (Table 1).

**Table 1:** Characteristics in patients with mild and severe CoA

	All (*n* = 53)	Mild (*n* = 26)	Severe (*n* = 27)	*P* value
Age (y)	18.15 ± 18.48	13.84 ± 18.71	22.30 ± 17.60	0.096
Male gender, n (%)	34 (64.2%)	14 (53.8%)	20 (74.1%)	0.158
BSA (m^2^)	1.06 ± 1.56	0.85 ± 0.50	1.26 ± 0.55	0.007
Hypertension, n (%)	19 (35.8%)	6 (23.1%)	13 (48.1%)	0.086
Simple CoA, n (%)	18 (34.0%)	7 (26.9%)	11 (40.7%)	0.387
Associated cardiovascular complications				
BAV, n (%)	9 (17.0%)	5 (19.2%)	4 (14.8%)	0.728
PDA, n (%)	16 (30.2%)	11 (42.3%)	5 (18.5%)	0.077
VSD, n (%)	15 (28.3%)	9 (34.6%)	6 (22.2%)	0.372
ASD, n (%)	3 (5.7%)	1 (3.8%)	2 (7.4%)	1.000
AR, n (%)	7 (13.2%)	2 (7.7%)	5 (18.5%)	0.42
AS, n (%)	5 (9.4%)	3 (11.5%)	2 (7.4%)	0.669
Collateral circulation, n(%)	21 (39.6%)	6 (23.1%)	15 (55.5%)	0.024
Z score of aorta				
Ascending aorta	2.26 ± 0.51	2.10 ± 0.57	2.41 ± 0.39	0.025
Pre-coarctation	1.85 ± 0.52	1.83 ± 0.45	1.87 ± 0.59	0.743
Aortic arch	1.47 ± 0.40	1.49 ± 0.45	1.45 ± 0.34	0.659
Post-coarctation	1.93 ± 0.52	1.68 ± 0.43	2.17 ± 0.48	< 0.001
Aortic dilation				
Ascending aorta	37 (69.8%)	14 (53.8%)	23 (85.2%)	0.018
Pre-coarctation	20 (37.7%)	9 (34.6%)	11 (40.7%)	0.779
Aortic arch	4 (7.5%)	3 (11.5%)	1 (3.7%)	0.351
Post-coarctation	20 (37.7%)	4 (15.4%)	16 (59.3%)	0.002

Values are showed as mean ± SD or count (percent)

*P* value stands for the difference between mild group and severe group

*Abbreviations: BSA* body surface area, *CoA* coarctation of aorta, *BAV* bicuspid aortic valve, *PDA* patent ductus arteriosus, *VSD* ventricular septal defect, *AR* aortic regurgitation, *AS* aortic valve stenosis

### Prevalence of aortic dilatation and aortic diameters

A total of 81 aortic dilations at different levels were developed in 40 (75.5%) patients and the most common site of dilation was the ascending aorta. Regarding the aorta, the z-scores of the ascending aorta and post-coarctation aorta were significantly higher in the severe group than in the mild group (2.10 ± 0.57 vs. 2.41 ± 0.39, and 1.68 ± 0.43 vs. 2.17 ± 0.48, respectively, both *p* < 0.05; Table 1). The severe group developed more dilation of the ascending aorta (85.2% vs. 53.8%, *p* < 0.05) and post-coarctation aorta (59.3% vs. 15.4%, *p* < 0.05) compared with the mild group.

### Aortic diameter or dilation and associated predictors

As for ascending aorta, degree of coarctation was associated with the z-score of the ascending aorta (*r* = − 0.356, *p* < 0.05). Ascending aortic dilation displayed a negative correlation with degree of coarctation (*r* = − 0.375, *p* = 0.006; Additional file 1).

The collateral circulation was associated with z-score of the ascending aorta (*r* = 0.375, *p* < 0.05). The occurrence of ascending aortic dilation showed a mild association with age (*r* = 0.308, *p* = 0.025) and collateral circulation (*r* = 0.281, *p* = 0.042; Additional file 1). Age was related to the z-score of ascending aorta, pre-coarctation aorta and aortic arch(r = 0.37–0.54, all *p* < 0.05).

Regarding post-coarctation aorta, degree of coarctation was related to the z-score of post-coarctation aorta (*r* = − 0.414, *p* < 0.05; Table 2). Degree of coarctation was mildly associated with post-coarctation aortic dilation((*r* = − 0.354, *p* < 0.05). In addition, there were mild relations between post-coarctation aortic dilation and age, AR, and VSD (*r* = 0.345, *r* = 0.386, and *r* = − 0.316, respectively, all *p* < 0.05; Additional file 2).

**Table 2:** Correlation between z score of aorta and degree of coarctation

	r	*P* value
Ascending aorta	−0.356	0.009
Pre-coarctation	−0.077	0.584
Aortic arch	0.064	0.647
Post-coarctation	−0.414	0.002

In univariate analysis, the risk of ascending aortic dilatation was significantly increased by age, severe coarctation, and collateral circulation (Table 3). The presence of post-coarctation aortic dilatation was associated with age, VSD, AR, and severity of coarctation (Table 4). In two multivariate models, increased severity of coarctation was identified as the independent risk factor of ascending aortic dilation (Odds ratio 7.46; 95% CI 1.19–46.76; *P* < 0.05, Nagelkerke R^2^ = 0.490, Table 3) and post-coarctation dilation (Odds ratio 8.42; 95% CI 1.84–38.56; *P* < 0.05, Nagelkerke R^2^ = 0.503, Table 4). No statistical association was found between the dilation of all levels of aorta and risk factors including gender, BSA, history of hypertension, BAV, PDA and AS (all *p* > 0.05).

**Table 3:** Univariate and multivariate analyses for the presence of ascending aortic dilation

Variable	Univariate analysis		Multivariate analysis	
	OR (95% CI)	*P* value	OR (95% CI)	*P* value
Age	–	0.0875	–	0.809
Gender (Female sex)	1.11 (0.33–3.74)	0.869	–	–
Hypertension	1.34 (0.38–4.67)	0.647	–	–
BAV	0.84 (0.18–3.87)	0.882	–	–
PDA	0.93 (0.26–3.32)	0.912	–	–
VSD	0.36 (0.10–1.25)	0.107	–	–
AR	2.90 (0.32–26.33)	0.343	–	–
AS	–	0.990	–	–
Collateral circulation	4.11 (1.00–16.84)	0.050	8.35 (0.85–82.11)	0.069
Severity of CoA(severe)	4.93 (1.33–18.31)	0.017	7.46 (1.19–46.76)	0.032

*Abbreviations: OR* odds ratio, *CI* confidence interval, *BAV* bicuspid aortic valve, *PDA* patent ductus arteriosus, *VSD* ventricular septal defect, *AR* aortic regurgitation, *AS* aortic valve stenosis, *CoA* coarctation of aorta

**Table 4:** Univariate and multivariate analyses for the presence of post-coarctation aortic dilation

Variable	Univariate analysis		Multivariate analysis	
	OR (95% CI)	*P* value	OR (95% CI)	*P* value
Age	–	0.705	–	–
Gender (male sex)	0.94 (0.30–3.01)	0.920	–	–
Hypertension	1.33 (0.42–4.21)	0.624	–	–
BAV	0.79 (0.18–3.61)	0.765	–	–
PDA	0.67 (0.19–2.31)	0.523	–	–
VSD	0.17 (0.03–0.86)	0.032	0.12 (0.01–0.80)	0.106
AR	0.73 (0.01–0.66)	0.020	0.04 (0.01–0.70)	0.080
AS	2.74 (0.42–18.01)	0.295	–	–
Collateral circulation	2.00 (0.64–6.23)	0.232	–	–
Severity of CoA(severe)	8.00 (2.15–29.74)	0.002	8.42 (1.84–38.56)	0.006

*Abbreviations: OR* odds ratio, *CI* confidence interval, *BAV* bicuspid aortic valve, *PDA* patent ductus arteriosus, *VSD* ventricular septal defect, *AR* aortic regurgitation, *AS* aortic valve stenosis, *CoA* coarctation of aorta

### Radiation dose

DSCT was conducted to avoid the excess exposure of radiation. Given that younger patients are sensitive to radiation, we calculated the estimated radiation dose within different age groups (Table 5). The mean effective dose was 1.69 ± 1.48 mSv in patients younger than 14 years old.

**Table 5:** Radiation dose estimation according to different age groups

	< 4 months	4 months-1 year	1-6 years	6–14 years	> 14 years
CDTI_vol_ (mGy)	6.86 ± 4.69	7.40 ± 6.06	11.82 ± 7.12	14.32 ± 7.87	28.91 ± 17.56
DLP (mGy·cm)	44.67 ± 33.38	47.09 ± 37.47	127.75 ± 73.25	179.71 ± 179.79	869.87 ± 741.10
ED (mSv)	1.74 ± 1.30	1.18 ± 1.00	2.30 ± 1.32	2.11 ± 2.18	6.31 ± 7.84

*Abbreviations: CTDIvol* volume CT dose index, *DLP* dose-length product, *ED* effective dose

## Discussion

This study demonstrated that aortic dilations presented commonly in ascending and post-coarctation aorta. Patients with increased age, presence of collateral circulation and severer coarctation were much easier to develop dilation of the ascending aorta. With increased age, severer coarctation, patients were much liable to be with post-coarctation dilation. Additionally, severity of coarctation was important predictor of the presence of ascending and post-coarctation aortic dilations.

In recent years, increased attention has been given to aortic dilation, which has been reported at various aortic levels [12]. Pathogenic factors including medial degeneration of the aorta, apoptosis of smooth muscle cells, elastic fiber fragmentation and hemodynamic factors were considered to lead to aortic dilation [2, 15]. Previous reports have demonstrated that valve dysfunction and age were associated with aortic dilation via TTE [2, 6, 16]. Nevertheless, which site has the biggest risk and what affects the dilation are controversial. Thus, DSCT was conducted to access the relevant risk factors of aortic dilation at different levels in patients with CoA.

Some studies have proven that the presence of coarctation is associated with the aortic wall complications [17]. Our data revealed that patients with a severe degree of coarctation were associated with dilation of the ascending aorta. This phenomenon was attributed to the stress difference in the aorta. Hemodynamic stress associated with coarctation might result in increased stroke volume that places increased wall stress on the ascending aorta and further leads to ascending aortic dilation [18–20]. Another reason for ascending aortic dilation is that severe coarctation induces the increase of afterload and elevates the blood pressure in the ascending aorta. Regarding the post-coarctation aorta, our results suggested that the degree of coarctation was associated with dilation of the post-coarctation aorta. This may be due to the increase in collateral blood flow and haemodynamic factors caused by the high velocity and turbulent flow downstream of the coarctation, or due to intrinsic character of the aortic wall [21, 22].

Collateral circulation functions as compensation of the aorta around the coarctation segment based on the increased blood pressure [22]. In our study, a significant connection was found between the presence of collateral circulation and dilation of the ascending aorta. This result can be explained by the increase in systemic arterial pressure and afterload when a patient with CoA presents ascending aortic dilation, which induces the formation of collateral circulation [22].

We confirmed that older patients were predictors of a larger ascending aorta and presence of ascending aortic dilation, which were consistent with previously reported results [12, 23]. This phenomenon is caused by the natural growth of the aorta and associated pathogenic factors, including medial necrosis and elastic fiber fragmentation, with increasing age [23–25].

Four variables showed no notably significant relation with aortic dilation. Firstly, we found no association between history of hypertension and aortic dilation and it was also reported by some other studies [17, 24]. This result may be account of the fact that the presence of aortic dilation could be caused by intrinsic factors except hemodynamic factors [15]. Secondly, presence of PDA didn’t show significant association with aortic dilation either, which might be explained by the irrelevance between PDA and blood volume. In addition, no association was found between BAV and aortic dilation in our study, inconsistent with what the previous reported that the BAV was a predictor of aortic complications including aortic dilation in adult patients with CoA [12, 17, 26]. However, limited number of patients were found with BAV in this group and the BAV patients’ age were relatively younger compared with the previous ones. Similarly but in agreement with the previous studies [27, 28], AS was also found with no notable relationship with aortic size. Thus, we believe a safe comment might be that further study was still needed to confirm the relationship between aortic valve complications and the aortic dilation in young CoA patients.

Recent years, dynamic cine-CT angiography can achieve a good detect of significant dynamic thoracic aortic motion with no added radiation or contrast exposure, which helps to evaluate the aorta more clearly [29]. Cardiac MRI and MRI angiography (MRA) have been applied widely for noninvasive morphological and functional evaluation of heart and blood vessels in patients with various congenital heart diseases including CoA, especially in serial follow-up after surgical repair or balloon angioplasty due to its free-from-radiation characteristic [30, 31]. Furthermore, edged cardiac MRI technology such as 4D-flow MRI, wins a place in major artery pre-operative planning, considering its qualitative and quantitative description of hemodynamic status surrounding the target aorta segment [32]. While however, relatively insufficient spatial resolution to display small vessels and high cost still limit its use in most of the medical centers, as well as on our patients in the past.

Aortic rupture is considered an important killer in patients with unrepaired CoA, and it usually develops via aortic dilation [2]. Our analysis suggested that risk factors might prompt aortic dilation in patients with CoA, especially for the ascending aorta and post-coarctation aorta, which may also aggravate the damage to the postoperative aorta. Therefore, appropriate preoperative evaluation of aortic dilation may assist in stratifying risks of patients with unrepaired CoA and serve as a reminder for clinical therapy. We should consider the degree of narrowing, collateral circulation, age and associated abnormalities in newly diagnosed CoA patients and reinforce the risk factor management to prevent aortic dilation and corresponding hazards.

There were several limitations to this study. First, due to the low prevalence of CoA and the strict inclusion and exclusion criterias, this retrospective single-center study only acquired limited data on patient characteristics, though the existing information was sufficient to conduct primary research. Second, although pediatric patients were exposed to radiation from CT, we used DSCT to decrease the radiation dose to clinically acceptable levels. Therefore, with a short examination time and extensive evaluation, DSCT is applicable for cardiovascular diseases. Third, a long-term follow up is necessary to evaluate the risk factors of progressive aortic dilation.

## Conclusions

In summary, aortic dilation of patients with CoA occurs primarily at the ascending and post-coarctation aorta. Combined with TTE, DSCT can comprehensively evaluate the degree of coarctation, collateral circulation and some other malformations and further perform an appropriate assessment of aorta for aiding in stratification of risk for aortic dilation in patients with CoA.

## Additional files

Additional file 1: Correlation between the occurrence of ascending aortic dilation and associated factors. (DOCX 16 kb)

Additional file 2: Correlation between the occurrence of post-coarctation aortic dilation and associated factors. (DOCX 22 kb)

## Abbreviations

AR: Aortic regurgitation
AS: Aortic valve stenosis
ASD: Atrial septal defect
BAV: Bicuspid aortic valve
BSA: Body surface area
CDR: Coarctation site-diaphragm ratio
CoA: Coarctation of aorta
CT: Computed tomography
DSCT: Dual-source computed tomography
MRA: Magnetic resonance angiography
MRI: Magnetic resonance imaging
PDA: Patent ductus arteriosus
TTE: Transthoracic echocardiography
VSD: Ventricular septal defect
