# Supplementary material for: Predictors of aortic dilation in patients with coarctation of the aorta: evaluation with dual-source computed tomography
Source: BMC Cardiovasc Disord. 2018 Jun 22;18:124. doi: 10.1186/s12872-018-0863-8 (PMC6013956; doi:10.1186/s12872-018-0863-8)
Supplement: Supplementary file 1 — Correlation between the occurrence of ascending aortic dilation and associated factors. (DOCX 16 kb) [file 12872_2018_863_MOESM1_ESM.docx]

**Additional file 1** Correlation between the occurrence of ascending aortic dilation and associated factors

|  | **r** | **P value** |
| --- | --- | --- |
| Age | 0.308 | 0.025 |
| Gender | -0.023 | 0.872 |
| BSA (m2) | 0.212 | 0.127 |
| Hypertension | 0.063 | 0.375 |
| Complexity of CoA | -0.124 | 0.672 |
| BAV | -0.031 | 0.826 |
| PDA | -0.015 | 0.914 |
| VSD | -0.226 | 0.104 |
| AR | 0.135 | 0.335 |
| AS | 0.212 | 0.127 |
| Collateral circulation | 0.281 | 0.042 |
| Degree of coarctation | -0.375 | 0.006 |

*Abbreviations:* BSA, [body](C:/Users/DEll/AppData/Local/youdao/dict/Application/6.3.69.8341/resultui/frame/javascript:void(0);) [surface](C:/Users/DEll/AppData/Local/youdao/dict/Application/6.3.69.8341/resultui/frame/javascript:void(0);) [area](C:/Users/DEll/AppData/Local/youdao/dict/Application/6.3.69.8341/resultui/frame/javascript:void(0);); CoA, coarctation of aorta; BAV, bicuspid aortic valve; PDA, [patent](C:/Users/DEll/AppData/Local/youdao/dict/Application/6.3.69.8341/resultui/frame/javascript:void(0);) [ductus](C:/Users/DEll/AppData/Local/youdao/dict/Application/6.3.69.8341/resultui/frame/javascript:void(0);) [arteriosus](C:/Users/DEll/AppData/Local/youdao/dict/Application/6.3.69.8341/resultui/frame/javascript:void(0);); VSD, ventricular septal defect; AR, [aortic](C:/Users/DEll/AppData/Local/youdao/dict/Application/6.3.69.8341/resultui/frame/javascript:void(0);) [regurgitation](C:/Users/DEll/AppData/Local/youdao/dict/Application/6.3.69.8341/resultui/frame/javascript:void(0);); AS, aortic valve stenosis.

Indication：Additional file 1 should be cited and placed at the results section of the manuscript (191 lines on page 9).
