# Supplementary material for: Predictors of aortic dilation in patients with coarctation of the aorta: evaluation with dual-source computed tomography
Source: BMC Cardiovasc Disord. 2018 Jun 22;18:124. doi: 10.1186/s12872-018-0863-8 (PMC6013956; doi:10.1186/s12872-018-0863-8)
Supplement: Supplementary file 2 — Correlation between the occurrence of post-coarctation aortic dilation and associated factors. (DOCX 22 kb) [file 12872_2018_863_MOESM2_ESM.docx]

**Additional file 2** Correlation between the occurrence of post-coarctation aortic dilation and associated factors

|  | **r** | **P value** |
| --- | --- | --- |
| Age | 0.345 | 0.011 |
| Male gender | -0.014 | 0.922 |
| BSA | 0.204 | 0.144 |
| Hypertension | 0.067 | 0.632 |
| Complexity of CoA | -0.099 | 0.480 |
| BAV | -0.041 | 0.770 |
| PDA | -0.088 | 0.531 |
| VSD | -0.316 | 0.021 |
| AR | 0.386 | 0.004 |
| AS | 0.148 | 0.289 |
| Collateral circulation | 0.165 | 0.237 |
| Degree of coarctation | -0.354 | 0.009 |

*Abbreviations:* BSA, [body](C:/Users/DEll/AppData/Local/youdao/dict/Application/6.3.69.8341/resultui/frame/javascript:void(0);) [surface](C:/Users/DEll/AppData/Local/youdao/dict/Application/6.3.69.8341/resultui/frame/javascript:void(0);) [area](C:/Users/DEll/AppData/Local/youdao/dict/Application/6.3.69.8341/resultui/frame/javascript:void(0);); CoA, coarctation of aorta; BAV, bicuspid aortic valve; PDA, [patent](C:/Users/DEll/AppData/Local/youdao/dict/Application/6.3.69.8341/resultui/frame/javascript:void(0);) [ductus](C:/Users/DEll/AppData/Local/youdao/dict/Application/6.3.69.8341/resultui/frame/javascript:void(0);) [arteriosus](C:/Users/DEll/AppData/Local/youdao/dict/Application/6.3.69.8341/resultui/frame/javascript:void(0);); VSD, ventricular septal defect; AR, [aortic](C:/Users/DEll/AppData/Local/youdao/dict/Application/6.3.69.8341/resultui/frame/javascript:void(0);) [regurgitation](C:/Users/DEll/AppData/Local/youdao/dict/Application/6.3.69.8341/resultui/frame/javascript:void(0);); AS, aortic valve stenosis.

Indication：Additional file 2 should be cited and placed at the results section of the manuscript (202 lines on page 10).
